# A Treatise on Spontaneous and Congenital Dislocations of the Hip, with a Description of a New Apparatus for Fracture of the Neck of the Thigh-Bone

**Published:** 1843-10

**Authors:** 


### Art. XVII.

*Ueber spontane und congenitale Luxationen, sowie über einen neuen Schenkelhalsbruch-Apparat.* Von J. Heine, Doctor der Medezin und Chirurgie, Gründer und Vorsteher der orthopädischen Heilanstalt zu Cannstatt.—*Stuttgart*, 1842. 8vo, pp. 84.

*A Treatise on Spontaneous and Congenital Dislocations of the Hip, with a description of a new Apparatus for Fracture of the Neck of the Thigh-bone.* By J. Heine, Doctor of Medicine and Surgery, Founder and Superintendent of the Orthopedic Institution at Cannstatt. —*Stuttgard*, 1842.

This essay contains an account of a bed invented by the author for the treatment of the affections of the lower limbs named in the title, and gives the results of the author's experience in relation to the chances of their relief or cure by its means. The style of the work is plain and practical; the contents are evidently the result of considerable experience; and the results arrived at highly creditable to the ingenuity and ability of the author. The applicability of the bed to fractures of the thigh is shown by a case communicated to the author by a friend, whilst its efficacy in affording whatever relief can be given in dislocations of the hip is amply illustrated by its employment in the numerous cases which have come under Dr. Heine's care.

The bed used by Dr. Heine consists of a firm framework, covered with a hard mattress, and furnished with means for the reception of the patient's evacuations. A strong girdle of leather, well padded, is passed round the pelvis, from the front of which two straps pass over the patient's groin and the perineum to two points on the back part of the girdle opposite their attachment in front. When this girdle is fixed, either to the mattress or by straps to the posts at the head of the bed, the parts above the hip-joints are necessarily fixed, and counter-extension can be conveniently made by force applied to the lower extremity. The lower extremity is laid on a splint, well protected by pads, and furnished with a shoe; to this shoe a screw is fixed, which, by its connexion with a rackwork fixed at the foot of the bed, can be lengthened or shortened, and thus draw the foot further from the pelvis, and thus necessarily lengthen the shortened limb. There are some other ingenious contrivances about the bed, but the general principle on which it is made will be sufficiently understood from this description.

The bed employed for fracture of the neck of the thigh-bone differs but little from that used for the reduction of dislocations; that employed for the former being on the whole the most simple, inasmuch as certain varieties of direction and elevation are occasionally necessary for the treatment of the variable direction which the dislocated limb may assume.

"Case i. John Koch, æt. 17, was exposed to cold, whilst heated with walking a considerable distance, in the autumn of 1838, and soon afterwards began to feel pain in his right hip-joint. The pain and lameness continued more or less till May, 1839, when the right limb was found to be short, for which reason he placed himself under medical care and got somewhat better; but as his ailments again returned, he applied to Dr. Heine, at about eight months from the commencement of his illness. The man appeared to be in moderately good health. There were no signs of inflammation about the joint, and very little pain even on forcible motion of the part. The limb was shortened considerably, and the head of the femur formed a moveable swelling on the dorsum ilii. Under these circumstances the patient was admitted into the hospital, and extension commenced in a few days, on June 25, 1839. The extension was applied at first very gradually and only for a short time, the patient being allowed to walk about during part of the day. On the 22d July, the head of the femur apparently slipped into the socket, but passed out again on relaxing the extension. On July 23d, the bone slipped into the socket, and assumed all its natural conditions of form and relation to the surrounding parts, except that of being three lines short, and having the trochanter major rather prominent. The patient was confined to bed now for three weeks, during which the limb was occasionally rotated on the apparatus. On the 20th of August, the patient was allowed to walk on crutches; the limb was found to be of the natural length; the rotation of the trochanter major more natural. The patient gradually left off his crutches, and left the hospital well, November 19th, wearing a belt round the hips. In December, 1841, Dr. Heine saw the patient, and found him able to walk six or eight hours daily; the two limbs were now the same length, and the stiffness in the joint gradually subsiding.

"Case ii. Philip Fischer, æt. 19, first felt pain in the left hip and knee during the summer of 1839, which continuing more or less till the spring of 1840, was then followed by shortening of the limb. On the 21st March, 1841, the limb presented the following conditions: The left limb was shortened one inch and half, whilst the head of the thigh bone could be felt quite moveable on the dorsum ilii. The patient was free from pain, and all inflammatory affection appeared to have ceased round the joint. Extension was applied gradually from the 6th of April, 1841, till May 8th, when the head of the femur slipped into the socket; the limb, however, remained three lines short and the trochanter somewhat prominent. The patient was treated as the first, and left the hospital, July 22d, 1841, able to walk well but rather clumsily. The leather belt round the hips was also here directed to be used.

Six months afterwards the patient walked with ease a distance requiring four hours to Dr. Heine, who detected no sign of lameness, and found both limbs equally strong.

"Case iii. Catharina Gauger, æt. 16, first felt pain in the right hip-joint in September, 1839, which continuing, was treated as disease of the joint. In January, 1840, the pain became less, but the limb became short. In May, 1841, the limb presented the following appearance: the right limb was small and shortened two inches. The head of the femur could be felt on the dorsum ilii. There was no pain, and all inflammation round the part appeared to have ceased. On May 20th, the extension was commenced. On the 10th of June, the extension had been made rather forcibly during the night, the patient was trying to relieve herself by drawing her body upwards, when something snapped audibly. On examining the limb, the two legs were the same length and the two trochanters on the same level. She was treated as the two previous cases, and left the hospital, walking easily in six months."

[Since this period Dr. Heine has received information that the lameness is hardly perceptible, that the limb continues to gain strength, and that she can walk quite well.]

"Case iv. Christopher Schreiber, æt. 16, first felt pain in the right hip-joint in September, 1841; the pain continued to increase and was attended with apparent elongation of the limb, to which shortening succeeded; increased by an accidental fall out of bed. In March, 1842, the limb presented the following appearances: the limb was shortened two Wirtemburg inches (of ten to the foot), and the head of the bone could be felt on the dorsum ilii, apparently fixed by the muscles and not surrounded by any thickening. The patient was free from pain, and all inflammation appeared to have ceased round the joint. Extension was commenced on the 14th April, 1842, and on the 13th May the head of the bone passed into the socket, the two limbs now being of the same length and the two trochanters on the same level. This patient was treated as the others, and at the usual period was allowed to walk about; the use of the limb gradually becoming more and more complete. This man was seized with symptoms of some obscure affection of the chest, and died on the 23d July, 1842. On examination a large tumour occupied the mediastinum, pressing on the heart and lungs.

"The right hip-joint was very accurately examined. The gluteus maximus and medius muscles were somewhat lax on their lower edges, whilst the gluteus minimus was considerably less firm and tense than in the natural condition of parts. The other muscles and parts round the joint presented nothing unusual, neither could any depressions in the muscles or remains of a false joint be discovered on the dorsum ilii. The capsule of the hip-joint was somewhat lax, but presented on its outer surface no morbid appearance, whilst the head of the femur was accurately fitted into the acetabulum. The head of the bone could with slight force be separated somewhat from the acetabulum, but could not be dislocated in any way. The hip-joint contained about a drachm and a half of a yellow fluid mixed with lymph. The synovial surface of the capsule, on the superior half of its circumference, was of a dark red colour and covered with a membrane of thickened tissue; no remains of any rent or laceration could however be found on any part of it. The ligamentum teres was long, thin, and of a dark colour; the glands of Havers were also dark-coloured and small. The head of the femur was about the natural size, covered with healthy cartilage, but marked with a few grooves. The head of the bone was however somewhat flattened instead of presenting its natural round form." (pp. 9-45.)

In addition to these cases, five others have been treated by Dr. Heine. The ages of these patients were respectively 5, 6, 9, 10, and 12 years; they all had suffered from disease of the joint for not less than two years, but had not suffered from abscesses. On the treatment being first employed, no inflammation of any importance remained; the head of the bone was drawn up on the dorsum ilii, and the limb was shortened one inch and half. In two cases, the head of the bone was moveable, and two of the patients could walk without crutches. So far as could be ascertained, the original disease appeared to depend on the previous strumous condition of the patient. In these cases extension was carefully but forcibly applied for some months, with some benefit to the shortening of the limb in two of the cases. In the other three, however, such pain was produced by extension, as to suggest the idea that adhesions had formed between the head of the femur and the surrounding parts.

From the result of these cases, Dr. Heine considers that the following rules may be laid down, as regulating the propriety of the employment of extension:

1. The removal of all inflammatory disturbance about the part is necessary before its employment.

2. The less advanced the disease is, the more favorable is the case for the employment of extension.

3. The existence of abscess precludes any attempts to return the bone to the joint.

4. The prominence and mobility of the great trochanter are favorable signs, as they indicate that the head of the bone is not adherent.

5. The prognosis is more favorable in affections of the hip from mechanical and rheumatic causes than from affections of the general health and scrofulous disease.

6. When extension is applied at an early period after the occurrence of the dislocation, it affords more chance of success than when it is applied at a later period.

The congenital dislocation of the hip has been observed by Dr. Heine in nine female and two male children, six of whom were affected in one hip and five in both. No decided benefit resulted in any of these cases from the employment of extension, as the bones returned to their previous position on the extension being omitted.

The account of a dissection of a congenital dislocation of the hip is recorded, and appears to be of sufficient interest to insert here.

"Frederic Schwarz, æt. 40, died of acute disease, having suffered all his life from lameness and deformity of the left hip. The trochanter major was situated one inch and a half higher than that of the opposite limb, the thigh and leg were both short, the patella situated more on the inner side of the joint than natural, and the foot turned slightly inwards. On dissection the patella was found to be united by fibrous substance to the inner condyle, whilst the muscles passing to the upper part of the tibia formed long fibrous threads. The muscles round the hip were extremely weak and small; the trochanter major was of its usual form, and the capsule firm and thick, with a smooth membrane lining it. The head of the femur was covered well with cartilage, somewhat flattened, and only half its natural size. The ligamentum teres was wanting, and its place of insertion into the femur was covered with cartilage. A smooth surface, situated above the natural position of the acetabulum, formed the articulating surface for the head of the femur, which was fixed to it by the firm capsule. Below was situated the natural acetabulum, of a narrow oval form, two lines in depth, and forming a cavity for the flattened trochanter minor." (p. 69.)

